# Supplementary material for: The health benefits and cost-effectiveness of complete healthy vending
Source: PLoS One. 2020 Sep 21;15(9):e0239483. doi: 10.1371/journal.pone.0239483 (PMC7505467; doi:10.1371/journal.pone.0239483)
Supplement: S1 Appendix — (DOCX) [file pone.0239483.s001.docx]

**Appendix 1: Hospital floor plan**

The A&E unit was isolated from all other sections of the hospital, with its own unique entrance. An alternative entrance nearby could be used to enter the main hospital site.

*Floor plan within A&E*


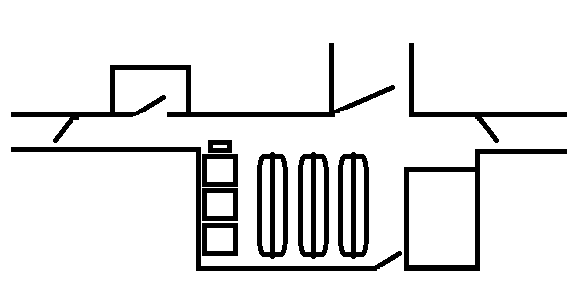


Office / reception

A&E entrance

Seating area

Vending machines (from left to right: water dispenser, coffee machine, snack machine, drink machine)

The reception was the main entrance to the maternity area of the hospital, but also provided alternative access to the main hospital site. A restaurant and shop could be accessed within approximately 25 metres of the vending machines.

*Floor plan within reception*

Alternative entrance to main hospital site

Corridor to restaurant and shop

Seating area

Vending machines (from top to bottom: snack machine, drink machine)

**
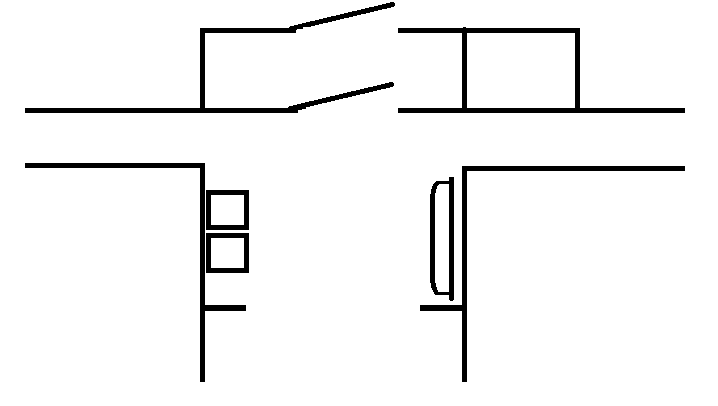
**
