# Supplementary material for: The health benefits and cost-effectiveness of complete healthy vending
Source: PLoS One. 2020 Sep 21;15(9):e0239483. doi: 10.1371/journal.pone.0239483 (PMC7505467; doi:10.1371/journal.pone.0239483)
Supplement: S2 Appendix — (DOCX) [file pone.0239483.s002.docx]

## **Appendix 2: Vending machine layout**

### Healthy Vending Layout

| #11  Walkers Baked Cheese + Onion | | #13  Walkers Baked Cheese + Onion | | #15  Walkers Baked Salt + Vinegar | | #17  Walkers Baked Ready Salted | |
| --- | --- | --- | --- | --- | --- | --- | --- |
| #21 French Fries Salt + Vinegar | | #23 French Fries Worcester Sauce | | #25 French Fries Ready Salted | | #27 French Fries Ready Salted | |
| #31 Popchips Sour Cream+ Onion | | #33 Popchips Sour Cream+ Onion | | #35 Popchips Sea Salt + Vinegar | | #37 Popchips Sea Salt + Vinegar | |
| #41 Popchips Ridges Smoky Bacon | | #43 Popchips Ridges Smoky Bacon | | #45 Popchips BBQ | | #47 Popchips BBQ | |
| #50  Polo  S/F | #51  Polo  S/F | #52  Nakd Peanut Delight | #53  Nakd Bakewell Tart | #54  Nakd Berry Delight | #55  Nakd Berry Delight | #56  Go Ahead Crispy Slices | #57  Go Ahead Crispy Slices |
| #60  Nakd Cashew Cookie | #61  Nakd Cashew Cookie | #62  Nakd Cocoa + Orange | #63  Nakd Cocoa + Orange | #64  Fruit + Nut Grab Bag | #65  Fruit + Nut Grab Bag | #66  Go Ahead Yogurt Breaks | #67  Go Ahead Yogurt Breaks |

### Unhealthy Vending Layout

| #11  Tyrrell’s Sea Salted | | #13  Tyrrell’s Sea Salted | | #15  Tyrrell’s Sea Salt+Vinegar | | #17  Tyrrell’s Sea Salt+Vinegar | |
| --- | --- | --- | --- | --- | --- | --- | --- |
| #21 Mini Cheddars BBQ | | #23 Mini Cheddars Original | | #25 Quavers Cheese | | #27 Quavers Cheese | |
| #31 Hula Hoops BBQ Beef | | #33 Hula Hoops BBQ Beef | | #35 McCoy’s Flame Grilled Steak | | #37 McCoy’s Flame Grilled Steak | |
| #41 Tyrrell’s  Mature Cheddar | | #43 Tyrrell’s  Mature Cheddar | | #45 Tyrrell’s  Roast Chicken | | #47 Tyrrell’s  Roast Chicken | |
| #50  Galaxy Caramel | #51  Galaxy Smooth Milk | #52  Kit Kat | #53  Kit Kat | #50  Twix | #51  Twix | #52  Maltesers | #53  Polo Mints |
| #60  Bounty | #61  Dairy Milk | #62  M&Ms Chocolate | #63  M&Ms Peanut | #60  Snickers | #61  Mars | #62  Bakewell Flapjack | #63  Chocolate Flapjack |
