# Supplementary material for: The health benefits and cost-effectiveness of complete healthy vending
Source: PLoS One. 2020 Sep 21;15(9):e0239483. doi: 10.1371/journal.pone.0239483 (PMC7505467; doi:10.1371/journal.pone.0239483)
Supplement: S3 Appendix — (DOCX) [file pone.0239483.s003.docx]

## Appendix 3. Nutritional information

Tables show totals per item for fat, saturated fat, carbohydrates, sugars and sodium.

### Healthy Range

| **Product number** | **Product** | **Kcals** | **Fat (g)** | **Saturated fat (g)** | **Carbohydrate (g)** | **Sugars (g)** | **Sodium (g)** |  |  |  |  |  |  |  |
| --- | --- | --- | --- | --- | --- | --- | --- | --- | --- | --- | --- | --- | --- | --- |
| 1 | French Fries Ready Salted | 91 | 3.4 | 0.3 | 13.7 | 0.2 | 0.53 |  |  |  |  |  |  |  |
| 2 | French Fries Salt & Vinegar | 92 | 3.4 | 0.3 | 13.9 | 0.3 | 0.48 |  |  |  |  |  |  |  |
| 3 | French Fries Worcester Sauce | 93 | 3.5 | 0.4 | 13.5 | 0.3 | 0.42 |  |  |  |  |  |  |  |
| 4 | Fruit Nut Grab Bag | 242 | 17.9 | 3.0 | 16.9 | 12.1 | 0.03 |  |  |  |  |  |  |  |
| 5 | Go Ahead Crispy Slice Apple | 165 | 3.0 | - | 32.7 | 13.8 | 0.27 |  |  |  |  |  |  |  |
| 6 | GoAhead Yogurt Breaks Forest Fruit | 142 | 3.6 | 1.6 | 25.8 | 12.4 | 0.18 |  |  |  |  |  |  |  |
| 7 | Nakd Berry Delight | 135 | 5.2 | 1.0 | 18.2 | 16.6 | 0.10 |  |  |  |  |  |  |  |
| 8 | Nakd Bakewell Tart | 137 | 5.9 | 1.2 | 17.6 | 16 | 0.10 |  |  |  |  |  |  |  |
| 9 | Nakd Cashew Cookie | 143 | 8.2 | 1.6 | 16.1 | 13.7 | 0.10 |  |  |  |  |  |  |  |
| 10 | Nakd Cocoa Orange | 145 | 7.0 | 1.5 | 15.8 | 13.6 | 0.10 |  |  |  |  |  |  |  |
| 11 | Nakd Peanut Delight | 149 | 7.5 | 1.4 | 14.6 | 13.6 | 0.20 |  |  |  |  |  |  |  |
| 12 | Polo Sugar Free | 78 | 0 | 0 | 32.9 | 0.1 | < 0.01 |  |  |  |  |  |  |  |
| 13 | Popchips Smoky Bacon | 99 | 3.5 | 0.3 | 15.0 | 0.8 | 0.34 |  |  |  |  |  |  |  |
| 14 | Popchips BBQ | 97 | 3.6 | 0.3 | 14.0 | 2.1 | 0.49 |  |  |  |  |  |  |  |
| 15 | Popchips Sour Cream & Onion | 95 | 3.4 | 0.4 | 15.0 | 1 | 0.45 |  |  |  |  |  |  |  |
| 16 | Popchips Salt & Vinegar | 95 | 3.3 | 0.3 | 15.0 | 0.6 | 0.50 |  |  |  |  |  |  |  |
| 17 | Walkers Baked Cheese & Onion | 163 | 5.1 | 0.5 | 25.7 | 2.8 | 0.35 |  |  |  |  |  |  |  |
| 18 | Walkers Baked Ready Salted | 163 | 5.1 | 0.5 | 25.9 | 2.2 | 0.44 |  |  |  |  |  |  |  |
| 19 | Walkers Baked Salt & Vinegar | 162 | 5.0 | 0.5 | 25.5 | 2.4 | 0.30 |  |  |  |  |  |  |  |
|  | | | | | | | | |  |  |  |  |  |  |
| Unhealthy range  \| **Product number** \| **Product** \| **Kcals** \| **Fat (g)** \| **Saturated fat (g)** \| **Carbohydrate (g)** \| **Sugars (g)** \| **Sodium (g)** \| \| --- \| --- \| --- \| --- \| --- \| --- \| --- \| --- \| \| 20 \| Bakewell Flapjack \| 450 \| 21.6 \| 12.0 \| 57.5 \| 28.9 \| 0.45 \| \| 21 \| Bounty \| 278 \| 14.6 \| 12.1 \| 33.6 \| 27.5 \| 0.14 \| \| 22 \| Chocolate Flapjack \| 438 \| 20.8 \| 9.5 \| 56.4 \| 18.2 \| 0.03 \| \| 23 \| Cadbury Dairy Milk \| 240 \| 14.0 \| 8.3 \| 25.7 \| 25.2 \| 0.06 \| \| 24 \| Galaxy Caramel \| 232 \| 11.0 \| 7.0 \| 30.0 \| 27.0 \| 0.08 \| \| 25 \| Galaxy Smooth Milk \| 229 \| 14.0 \| 8.0 \| 23.0 \| 23.0 \| 0.05 \| \| 26 \| Hula Hoops BBQ Beef \| 172 \| 8.8 \| 0.9 \| 21.0 \| 0.5 \| 0.80 \| \| 27 \| Kit Kat \| 209 \| 10.2 \| 5.7 \| 26.1 \| 21.3 \| 0.10 \| \| 28 \| Maltesers \| 186 \| 9.1 \| 5.6 \| 23.0 \| 19.0 \| 0.17 \| \| 29 \| Mars \| 230 \| 8.6 \| 4.2 \| 35.3 \| 30.5 \| 0.20 \| \| 30 \| McCoy's Flame Grilled Steak \| 250 \| 15.0 \| 1.3 \| 25.0 \| 1.1 \| 0.71 \| \| 31 \| Mini Cheddars BBQ \| 262 \| 15.0 \| 6.0 \| 26.0 \| 2.0 \| 0.32 \| \| 32 \| Mini Cheddars Original \| 256 \| 14.6 \| 5.8 \| 25.0 \| 2.6 \| 1.20 \| \| 33 \| M&M's Milk Chocolate \| 218 \| 9.2 \| 5.7 \| 31.0 \| 30.0 \| 0.05 \| \| 34 \| M&M's Peanut \| 230 \| 11.4 \| 4.6 \| 26.6 \| 24.1 \| 0.04 \| \| 35 \| Polo \| 137 \| - \| - \| 33.4 \| 32.5 \| <0.01 \| \| 36 \| Quavers \| 182 \| 10.0 \| 0.9 \| 21.0 \| 0.9 \| 0.73 \| \| 37 \| Snickers \| 245 \| 13.4 \| 4.7 \| 26.2 \| 21.7 \| 0.21 \| \| 38 \| Twix \| 248 \| 12 \| 7.0 \| 32.2 \| 24.4 \| 0.22 \| \| 39 \| Tyrrells Mature Cheddar & Chives \| 179 \| 9.2 \| 1.1 \| 21.5 \| 1.2 \| 0.50 \| \| 40 \| Tyrrells Roast Chicken \| 180 \| 9.2 \| 1.1 \| 21.0 \| 2.3 \| 0.40 \| \| 41 \| Tyrrells Sea Salt \| 196 \| 10.9 \| 1.2 \| 20.7 \| 0.2 \| 0.32 \| \| 42 \| Tyrrells Sea Salt & Cider Vinegar \| 195 \| 10.4 \| 1.1 \| 22.4 \| 0.7 \| 0.70 \| | | | | | | | | |  |  |  |  |  |  |
|  | | | | | | | | |  |  |  |  |  |  |
|  | | | | | | | | |  |  |  |  |  |  |
|  | | | | | | | | |  |  |  |  |  |  |
