# Supplementary material for: The health benefits and cost-effectiveness of complete healthy vending
Source: PLoS One. 2020 Sep 21;15(9):e0239483. doi: 10.1371/journal.pone.0239483 (PMC7505467; doi:10.1371/journal.pone.0239483)
Supplement: S4 Appendix — (DOCX) [file pone.0239483.s004.docx]

## Appendix 4. Product Cost and Profitability

Tables show wholesale cost and profit margins for each product on offer. All prices are in British Pound Sterling (GBP). The purchase price for each product was 80p.

### Healthy Range

| **Product number** | **Product** | | | | **Wholesale cost per item (£)** | **Profit margin per item (£)** |
| --- | --- | --- | --- | --- | --- | --- |
| 1 | French Fries Ready Salted | | | | 0.40 | 0.40 |
| 2 | French Fries Salt & Vinegar | | | | 0.40 | 0.40 |
| 3 | French Fries Worcester Sauce | | | | 0.40 | 0.40 |
| 4 | Fruit Nut Grab Bag | | | | 0.42 | 0.38 |
| 5 | Go Ahead Crispy Slice Apple | | | | 0.44 | 0.36 |
| 6 | GoAhead Yogurt Breaks Forest Fruit | | | | 0.44 | 0.36 |
| 7 | Nakd Berry Delight | | | | 0.45 | 0.35 |
| 8 | Nakd Bakewell Tart | | | | 0.45 | 0.35 |
| 9 | Nakd Cashew Cookie | | | | 0.45 | 0.35 |
| 10 | Nakd Cocoa Orange | | | | 0.45 | 0.35 |
| 11 | Nakd Peanut Delight | | | | 0.45 | 0.35 |
| 12 | Polo Sugar Free | | | | 0.38 | 0.42 |
| 13 | Popchips Smoky Bacon | | | | 0.47 | 0.33 |
| 14 | Popchips BBQ | | | | 0.47 | 0.33 |
| 15 | Popchips Sour Cream & Onion | | | | 0.47 | 0.33 |
| 16 | Popchips Salt & Vinegar | | | | 0.47 | 0.33 |
| 17 | Walkers Baked Cheese & Onion | | | | 0.45 | 0.35 |
| 18 | Walkers Baked Ready Salted | | | | 0.45 | 0.35 |
| 19 | Walkers Baked Salt & Vinegar | | | | 0.45 | 0.35 |
|  | |  |  |  |  |  |
|  | |  |  |  |  |  |
|  | |  |  |  |  |  |
|  | |  |  |  |  |  |
|  | |  |  |  |  |  |

### Unhealthy Range

| **Product number** | **Product** | | | | **Wholesale cost per item (£)** | **Profit margin per item (£)** |
| --- | --- | --- | --- | --- | --- | --- |
| 20 | Bakewell Flapjack | | | | 0.42 | 0.38 |
| 21 | Bounty | | | | 0.46 | 0.34 |
| 22 | Chocolate Flapjack | | | | 0.42 | 0.38 |
| 23 | Cadbury Dairy Milk | | | | 0.48 | 0.32 |
| 24 | Galaxy Caramel | | | | 0.50 | 0.30 |
| 25 | Galaxy Smooth Milk | | | | 0.50 | 0.30 |
| 26 | Hula Hoops BBQ Beef | | | | 0.30 | 0.50 |
| 27 | Kit Kat | | | | 0.44 | 0.36 |
| 28 | Maltesers | | | | 0.48 | 0.32 |
| 29 | Mars | | | | 0.43 | 0.37 |
| 30 | McCoy's Flame Grilled Steak | | | | 0.44 | 0.36 |
| 31 | Mini Cheddars BBQ | | | | 0.40 | 0.40 |
| 32 | Mini Cheddars Original | | | | 0.40 | 0.40 |
| 33 | M&M's Milk Chocolate | | | | 0.51 | 0.29 |
| 34 | M&M's Peanut | | | | 0.51 | 0.29 |
| 35 | Polo | | | | 0.35 | 0.45 |
| 36 | Quavers | | | | 0.42 | 0.38 |
| 37 | Snickers | | | | 0.43 | 0.37 |
| 38 | Twix | | | | 0.41 | 0.39 |
| 39 | Tyrrells Mature Cheddar & Chives | | | | 0.43 | 0.37 |
| 40 | Tyrrells Roast Chicken | | | | 0.43 | 0.37 |
| 41 | Tyrrells Sea Salt | | | | 0.43 | 0.37 |
| 42 | Tyrrells Sea Salt & Cider Vinegar | | | | 0.43 | 0.37 |
|  | |  |  |  |  |  |
|  | |  |  |  |  |  |
|  | |  |  |  |  |  |
|  | |  |  |  |  |  |
|  | |  |  |  |  |  |
