# Supplementary material for: The health benefits and cost-effectiveness of complete healthy vending
Source: PLoS One. 2020 Sep 21;15(9):e0239483. doi: 10.1371/journal.pone.0239483 (PMC7505467; doi:10.1371/journal.pone.0239483)
Supplement: S5 Appendix — (DOCX) [file pone.0239483.s005.docx]

## Appendix 5. Nutritional guidelines for vending.

Welsh Government’s healthy vending directive guidelines were used to determine which products were to be classified as healthy or unhealthy. All criteria discussed below come from the Welsh Government document detailing these guidelines [1]. The guidelines indicate that all vending snacks sold in Welsh hospitals must be the healthier option within its product range. This appendix serves as a summary of key components of the guidelines that have relevance to our study.

**Nutrient levels**

Products were assessed against specific criteria detailed in the guidelines [1]. Products were determined to have high levels of a nutrient if its value was greater than those listed in the table below:

Table 1. Highest level of each nutrient allowed by the guidelines per product.

| **Nutrient** | **Highest level allowed per 100g (g)** | **Highest level allowed per portion (g)** |
| --- | --- | --- |
| Fat | 20 | 21 |
| Saturated Fat | 5 | 6 |
| Added sugars | 12.5 | 15 |
| Salt | 1.5 | 2.4 |

**Exemptions**

The guidelines note that high levels of one or more of the nutrients are permitted for certain food categories [1], to allow more flexibility in sourcing healthier alternatives. Details of the product categories sold in the experiment with specific exemptions are available below:
*Biscuits and crackers e*.g. Plain biscuits, crackers, rice cakes, oatcakes.
Criteria: Can be vended even if one of the four nutrients is classed as high.

*Crisps and savoury snacks e*.g. Fried potato products, baked crisps, pretzels.
Criteria: High for salt permitted.

*Nuts and seeds e*.g. Coated/roasted nut and seed snacks, and those without added salt/sugar.
Criteria: High for fat and/or saturated fat.

**References**

1. Welsh Government. Health promoting hospital vending guidance [Internet]. 2012 [cited 2020 Apr 13]. Available from: <https://gov.wales/sites/default/files/publications/2019-07/directions-to-local-health-boards-in-wales-and-velindre-national-health-service-trust-2012-no-5-guidance.pdf>
