# Supplementary material for: The health benefits and cost-effectiveness of complete healthy vending
Source: PLoS One. 2020 Sep 21;15(9):e0239483. doi: 10.1371/journal.pone.0239483 (PMC7505467; doi:10.1371/journal.pone.0239483)
Supplement: Attachment — Submitted filename: Response to Reviewers.docx [file pone.0239483.s006.docx]

July 15^th^, 2020

Dear Zhifeng Gao,

Thank you for reviewing our manuscript, “The health benefits and cost-effectiveness of complete healthy vending.” We are grateful to you and the reviewers for your time and for sharing your insight. The manuscript has been edited to address your concerns.

As well as the changes required to fit the stylistic requirements of the journal, we have added some depth and clarity to the narrative in the General Discussion, and have shed some more light on the nutritional guidelines used to categorize the products used. We have also included the DOI for accessing our data. A detailed response to all the concerns raised by the reviewers can be viewed below.

Kind regards,

Melda Lois Griffiths
PhD Candidate at Cardiff University School of Psychology

On behalf of all authors.

**Changes made in response to listed journal requirements**

- PLOS ONE’s style requirements, including those for file naming, have now been met:
  - Formatting of headings
  - Figure citations and captions corrected (and Fig 4 now referred to in-text)
  - Funding information removed from acknowledgements
  - Supporting information captions added to the end of the manuscript, all in-text citations have been updated to the correct format, and file names have been changed to the correct format
- The final dataset is available on the Open Science Framework, DOI 10.17605/OSF.IO/H2ZJ6

**Changes made in response to reviewer comments**

**Reviewer 1**

Comment:
*“1. What do the results actually show us? In other words what is the substantive contribution to the field?
Does not the paper essentially show that if (mildly) hungry people face an assortment they will select an option that best fits their preference structure (or refrain from buying if it is not there). This, by definition, will lead to fewer and lower caloric choices in the healthy assortment condition. This is because, assuming that preferences are, on average, biased towards less healthy options, it is less likely that a preferred option is available and if it is, in the healthy assortment, it will by definition lead to reduced caloric intake.

It would be worthwhile if you provide such deeper reflection in the discussion section beyond what you now state on page 18: “Clearly, if the primary aim of an intervention is to increase healthy behaviour, completely replacing regular products with healthy products has the greatest impact”.”*

Response:

We have altered the manuscript to make the primary contribution clearer. We argue that complete healthy vending does not by definition lead to lower caloric consumption overall because individuals may have engaged in compensatory behaviors to make up for the lower calorie count of the healthy products. Our data demonstrates that this did not happen. Furthermore, while sales were lower in the healthy condition (although not significantly so), the drop was surprisingly small. Consumers seem relatively flexible in what they will purchase from a vending machine: if their regular products are not present, they simply purchase something else, even if it is a healthy product.

Comment:
*“2. As your data are at vending machine level, it is clear that you do not have access to individual level purchases. But one rival explanation could be that “snack preferences are heterogenous across population”, “people choose according to their product preferences”, and “different vending machine assortments attract different consumer segments”. Essentially your paper would then indicate that the segment of “calorie-lovers” is larger than that of “health motivated consumers”. Maybe this is an explanation you could also discuss as your results also show that these impulses are not strong enough for individual to make an effort to obtain the product from elsewhere if it is not available in the vending machine. Related to this point, you do unfortunately not report on the price levels of the items (only the margin which is surprisingly similar across healthy and unhealthy items?). But, another alternative explanation could be that higher priced assortments (which I assume the healthy options will be) appeal to a different (and smaller) consumer segment. Could you please reflect on the prices and reveal them in the table in supplementary materials?”*

Response:

This is a very interesting idea. We have added a paragraph discussing it in the GD.

We agree that different priced products will appeal to different consumer segments, and to control for this, we priced all products at 80p. This was originally noted in the “Vending snacks” section of the methods. We have now further emphasized the rationale behind our pricing in this section, and have added product cost and profitability information for all products in S4 Appendix.

Comment:
*“3. Also I think that the discrepancy between significantly fewer purchases, but non-significant loss in profit (despite the fact that average margin is similar (42 p) across the two conditions, is not very convincing. It is not fully clear to me how you relate this to “heterogeneity” as an explanation. That could use some more discussion, also in managerial terms as I assume that the relevant commercial benefit for the hospital is at vending machine level, not item level?”*

Response:

We found the same pattern of results for sales volume and profit, contrary to the reviewer’s comment above. We have now made this clearer in the discussion section.

The commercial benefit for the hospital is at the overall profit level, not the item level, but we are not sure why the reviewer has this concern.

Comment:
*“4. The other issue is the external validity of your findings that could use some further elaboration. With a 30% drop in profit and the large across item variability in sales (both for healthy and unhealthy category by the way) illustrated in your figure 5 what would be key managerial implications for vending machine managers? Could you reflect on whether such “all healthy” business model could be attainable for them? This could use more in depth discussion in the discussion section.”*

Response:

We have added a new section, “Implications for vendors”, that helps address this query.

**Reviewer 2**

Comment:

*“One concern that I have is related to the nutritional guidelines to determine whether a product is relatively healthier or not. These criteria are 'best in product class' criteria, which means that they might be not that strict. It would be helpful to provide more information and examples of products that do and do not meet the criteria. Are the Welsh Hospital Healthy Vending directive constraints developed in cooperation with the food or catering industry? How are they different from other nutritional standards such as those developed for schools?”*

Response:

We have altered the manuscript to provide more clarity on the nutrients targeted in the guidelines, and have highlighted that they were developed by the Welsh government. More details are now accessible in S5 Appendix, which lists the maximum level of fat, saturated fat, added sugars and salt allowed in any product, and any exemptions that may apply. We have also made reference to the generalizability of the results to school settings which already have their own, arguably stricter, guidelines in place.
